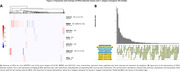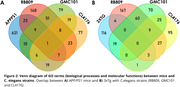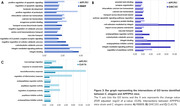# Shared transcriptomic similarities between mouse and *C. elegans* transgenic models of Alzheimer’s disease

**DOI:** 10.1002/alz.092954

**Published:** 2025-01-03

**Authors:** Vitória Vitalina Margarita Ryssina, Marco De Bastiani, Flávia Suelen De Oliveira Pereira, Giovanna Carello‐Collar, Karine Rigon Zimmer, Eduardo R. Zimmer

**Affiliations:** ^1^ Universidade Federal de Ciências da Saúde de Porto Alegre, Porto Alegre, Rio Grande do Sul Brazil; ^2^ Universidade Federal do Rio Grande do Sul, Porto Alegre, Rio Grande do Sul Brazil; ^3^ Universidade Federal do Pampa, Uruguaiana, Rio Grande do Sul Brazil; ^4^ Universidade Federal do Rio Grande do Sul, Porto Alegre, RS Brazil; ^5^ Universidade Federal de Ciencias da Saude de Porto Alegre, Porto Alegre, Rio Grande do Sul Brazil

## Abstract

**Background:**

The molecular mechanisms associated with Alzheimer’s Disease (AD) have been extensively studied in mouse models (*Mus musculus)*. However, experimental research in these models is costly and time‐consuming. In this context, the nematode *Caenorhabditis elegans* (*C. elegans*) is an interesting alternative model for studying AD. Nonetheless, whether *C. elegans* and mouse models of AD share transcriptomic similarities remains unclear. Thus, this study aims to investigate the transcriptomics overlap between *C. elegan*s and *M. musculus* AD models.

**Method:**

We obtained hippocampus and whole‐body transcriptomics data, respectively, from transgenic strains of AD mice (APP/PS1 and 3xTg) and *C. elegans* (RB809, GMC101, and CL4176) from the NCBI GEO database. The nematode strain RB809 presents a mutation in the microtubule‐associated protein homolog to human *Tau*. GMC101 and CL4176 strains aggregate the amyloid‐beta peptide (Aβ). We performed differential expression analysis (DEA) (FDR‐adjusted *p‐value < 0.05*) using the DESeq2 and Limma package to evaluate the differentially expressed genes (DEGs). Additionally, we conducted a functional enrichment analysis (FEA) to obtain Gene ontology of Biological Processes (GOBP) and Molecular Function (GOMF) terms (*p‐value < 0.01*).

**Result:**

The pattern of gene expression can be visualized in **Figure 1A. All nematode strains exhibited greater similarity with APP/PS1 mice compared to the 3xTg. Specifically, 94 DEGs were identified as shared between APP/PS1 mice and nematode strain RB809 (Figure 1B**)**. Concerning GMC101 and CL4176 strains, 41 and 12 DEGs were found shared, respectively. FEA analysis revealed GOBP terms shared among models involved in similar biological processes (Figure 2). Notably, “Aβ binding”, “calcium ion homeostasis”, “neuroinflammatory** response”, and “regulation of inflammatory response” (**Figure 3**) were identified.

**Conclusion:**

Our results indicate significant overlaps in genes and biological processes associated with AD and neurodegeneration between AD nematode and mice models, highlighting the translational conservation of fundamental pathological mechanisms. The differences in expression and processes between the various strains of *C. elegans* and the mouse models offer a unique window to investigate specific molecular aspects of AD.